# EXPLORING THE FREQUENT VISITS AND ASSOCIATED FACTORS OF OLDER ADULTS VISITING EMERGENCY ROOM

**DOI:** 10.1093/geroni/igae098.2915

**Published:** 2024-12-31

**Authors:** Ying-Chun Li, Po-Yen Chang, Wang-Chuan Juang, Yu-Qi Cheng

**Affiliations:** National Sun Yat-sen University, Kaohsiung, Kaohsiung, Taiwan (Republic of China); National Sun Yat-sen University, Kaohsiung, Kaohsiung, Taiwan (Republic of China); Kaohsiung Veterans General Hospital, Kaohsiung Veterans General Hospital, Kaohsiung, Taiwan (Republic of China); National Sun Yat-sen University, Kaohsiung, Kaohsiung, Taiwan (Republic of China)

## Abstract

Older adults may frequently visit the emergency department (ED) due to numerous comorbidities. Studies pointed out that frequent ED visits are one of the main reasons for emergency overcrowding which will affect the service capability of medical institutions. Therefore, this study mainly explored the risk factors of frequent ED visits by older adults. A retrospective study with an ED database from a medical center in southern Taiwan collected older than 65 years of adults who visited the ED at least once in 2019 and 2020. They were classified into frequent users with more than four ED visits per year. The propensity-score-matching was used in a 1:1 ratio to compare frequent ED users to the control group. Research results indicated that male, veterans, triage 3 status, summer, and day shifts visited the ED more frequently (p< 0.01). Multivariate logistic regression results showed that those who entered the ED on their own and were admitted for digestive, genitourinary systems, and factors influencing health status had a higher odds ratio of visiting the ED frequently. Negative binomial regression results presented that frequent ED users will have longer hospital stays than those non-ED frequent users. In conclusion, frequent ED users consume more medical resources thus may lead to ED overcrowding. We suggest that hospitals and physicians should pay more attention to this group. Through efficient policy advocacy and disease prevention to improve the quality of care for older adults.

